# Screening of Rhizosphere Bacteria and Nematode Populations Associated with Soybean Roots in the Mpumalanga Highveld of South Africa

**DOI:** 10.3390/microorganisms9091813

**Published:** 2021-08-26

**Authors:** Gerhard Engelbrecht, Sarina Claassens, Charlotte M. S. Mienie, Hendrika Fourie

**Affiliations:** Unit for Environmental Sciences and Management, North-West University, Private Bag X6001, Potchefstroom 2520, South Africa; Sarina.Claassens@nwu.ac.za (S.C.); Charlotte.Mienie@nwu.ac.za (C.M.S.M.); Driekie.Fourie@nwu.ac.za (H.F.)

**Keywords:** bacteria, biological control, *Meloidogyne*, *Pratylenchus*, soybean

## Abstract

Soybean is among South Africa’s top crops in terms of production figures. Over the past few years there has been increasingly more damage caused to local soybean by plant-parasitic nematode infections. The presence of *Meloidogyne* (root-knot nematodes) and *Pratylenchus* spp. (root lesion nematodes) in soybean fields can cripple the country’s production, however, little is known about the soil microbial communities associated with soybean in relation to different levels of *Meloidogyne* and *Pratylenchus* infestations, as well as the interaction(s) between them. Therefore, this study aimed to identify the nematode population assemblages and endemic rhizosphere bacteria associated with soybean using Next Generation Sequencing (NGS). The abundance of bacterial genera that were then identified as being significant using linear discriminant analysis (LDA) Effect Size (LEfSe) was compared to the abundance of the most prevalent plant-parasitic nematode genera found across all sampled sites, *viz*. *Meloidogyne* and *Pratylenchus*. While several bacterial genera were identified as significant using LEfSe, only two with increased abundance were associated with decreased abundance of *Meloidogyne* and *Pratylenchus*. However, six bacterial genera were associated with decreased *Pratylenchus* abundance. It is therefore possible that endemic bacterial strains can serve as an alternative method for reducing densities of plant-parasitic nematode genera and in this way reduce the damages caused to this economically important crop.

## 1. Introduction

Plant parasitic nematodes (PPN) cause substantial yield losses to agricultural crops, with annual global crop losses estimated at $78 billion [1]. *Aphelenchoides bessseyi*, *Bursaphelenchus xylophilus*, *Ditylenchus dispaci*, *Globodera* spp., *Heterodera* spp., *Meloidogyne* spp., *Naccobus aberrans, Radopholus similis*, *Rotylenchulus reniformis* and *Xiphinema index* are considered the top 10 nematode pests worldwide [2]. Due to their global distribution and wide range of host plants, of all the PPN genera and species, root-knot nematodes (RKN; *Meloidogyne* spp.) and lesion nematodes (*Pratylenchus* spp.) are particularly harmful to crops in South Africa and can cause substantial damage and adversely affect production figures of a wide range of economically important crops, such as the potato, grain, oilseed, industrial and fruit crops produced in this country [3,4]. Of all *Meloidogyne* spp. documented to parasitize crops on a global scale, 22 are reported to occur in Africa [5], while 14 *Meloidogyne* spp. and 10 *Pratylenchus* spp., respectively, have been listed for South Africa [6,7,8,9].

In the Mpumalanga Highveld region of South Africa crops that are usually planted include maize (*Zea mays*), wheat (*Triticum* spp.), groundnut (*Arachis hypogaea*), soybean (*Glycine max* (L.) Merr.), sunflower (*Helianthus* spp.) and potato (*Solanum tuberosum*) [10] of which all are known hosts of both RKN and lesion nematodes. Of these crops, soybean is considered an important crop in South Africa, with the Mpumalanga Highveld region being one of the most important production regions [11]. Soybean is one of the most important summer legumes produced worldwide and serves as an important dietary protein and oil source for both animal and human consumption [12,13]. A major benefit of growing soybean is its ability to fix nitrogen, providing an environmentally friendly alternative to synthetic nitrogen application [13]. South African soybean production dates back to the 1960s when production was only 2631 metric tons (MT) [14]. Production of the crop drastically increased since then and during the 2019/2020 growing season the area planted to soybean were estimated at 705,000 hectares (ha) from which 1,245,500 MT seeds were produced. During the following season (2020/2021), South Africa experienced a record crop production, represented by 827,100 ha planted and 1,793,650 MT of seeds harvested [11]. With the local increase in, and expansion of soybean and maize production since the beginning of the century, the risk of the crop being infected by a wide range of diseases and pests was expected [15,16]. The continuous generation of knowledge regarding nematode pests associated with soybean and maize is hence crucial.

Since these crops is usually grown in warmer climates, PPN such as *Meloidogyne* and/or *Pratylenchus* are the genera that cause major damage to these crops [13]. Soybean roots infected by *Meloidogyne* are usually distinguished by the formation of galls which interfere with several root functions, including water uptake, while roots infected by *Pratylenchus* can be characterized by the formation of necrotic root tissues. These nematode pests also cause various above ground symptoms like stunted growth and reduced leaf size [4,9]. Apart from nematodes, soybean production is also impacted by microorganisms such as bacteria that are present in the soil. Bacteria like Bradyrhizobium or Rhizobium are applied as a standard practice to increase the nitrogen fixation of soybean while other bacterial genera such as *Bacillus* have the potential to reduce nematode densities and resultant damage due to their nematicidal activities. Chemical nematicides remain one of the most used methods in nematode management, yet increasingly more research is being done to identify and develop eco-friendly products by using bacteria with nematicidal potential [9]. This study was performed in stages, by firstly stratifying the two top PPN in the localities into high, medium and low according to their abundance. Secondly, the bacterial community structure in these strata were determined. Therefore, the aims of this study were: (1) to determine the PPN communities and bacterial rhizosphere communities associated with soybean grown in the Highveld region of South Africa, which is the second biggest local production area for the crop [11] and (2) to determine whether a potential biocontrol link exists between these endemic rhizosphere bacteria and the prevailing PPN communities.

## 2. Materials and Methods

### 2.1. Site Description

**Figure 1:**
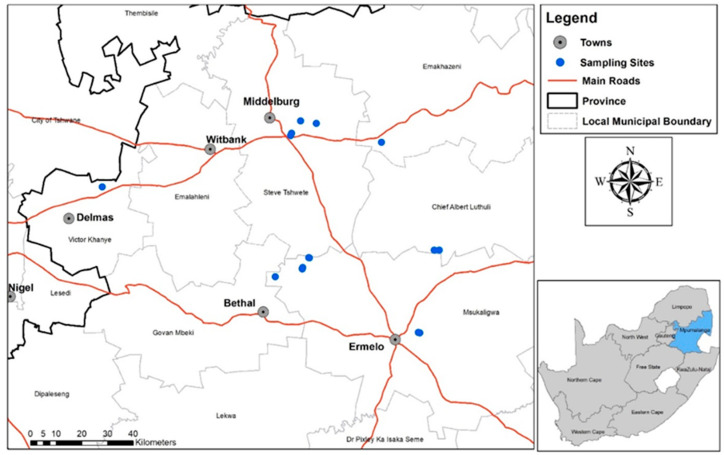
Soybean localities, situated in the Mpumalanga province of South Africa, where rhizosphere samples were obtained for nematode and microbe analyses during flowering of the crops in the 2019 summer growing season. (Illustration: Wiltrud Durand, BFAP, GIS & Crop Modelling).

South Africa is situated between the 22 and 35° S latitudes in the southern hemisphere and is characterized by diverse climatic conditions when compared to most sub-Saharan African countries. Located in Mpumalanga, one of the nine provinces of the country, the Mpumalanga Highveld (where this study was conducted) has a mean annual rainfall of 800–900 mm and an annual temperature range of 6–30 °C [10]. The grassland biome of this province, which contains rich and fertile upper layers, together with its annual rain and wide temperature ranges, makes it suitable for cultivation of crops such as soybean. In the 2019 summer growing season, rhizosphere samples (soil and roots) were taken from 15 fields where soybean was grown in the Mpumalanga Highveld of South Africa (Figure 1).

**Figure 2:**
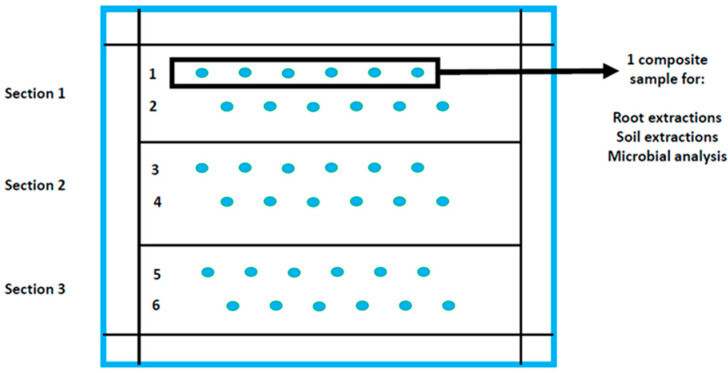
Sampling strategy at each of the 15 soybean localities sampled during the 2019 growing season for nematode and microbe analyses (Illustration: Gerhard Engelbrecht, North-West University). Each field therefore had a total of 6 composite root and soil samples that were analyzed.

The fields were spread across the province as seen in Supplementary Table, from locations located between 1518–1747 m above sea level with maize-soybean rotations being practiced. Each field was divided into three sections depending on the size of the locality (Figure 2). Sampling of roots and soil was done in a W shape in each section and the distance between points differed in size according to the size of the locality. Therefore, in each section two rows were selected where the roots and soil (approximately 30 g of soil around the root per plant) of 6 soybean plants were sampled per row. The root samples of each row were cut up into 1 cm pieces, pooled and homogenized before being used for nematode analyses. Six soil samples collected in each row were also pooled and homogenized, of which 50 g was taken for microbial analyses. This was done for 6 rows (2 rows per section) per field.

### 2.2. Extraction of PPN from Soybean Roots

Nematodes were extracted from 20 g of composite root samples, for each of the fields using the adapted centrifugal-flotation method [17] and transferred to a De Grisse counting dish [18]. The nematodes were counted and concurrently identified to the genus level using an ECLIPSE TS100 inverted microscope (Nikon Corporation, Tokyo, Japan) at 40× magnification.

### 2.3. DNA Extraction of Microbial Communities from the Soil

To extract the DNA of microbial communities from the composite soil samples, 0.25 g of each composite sample was used (Figure 2). This was done by using the NucleoSpin^®^ Soil kit (Macherey-Nagel, Düren, Germany) with the optimal lysis buffer system (a combination of SL 2 and Enhancer SX). The concentration of the extracted microbial DNA (absorbance at 260 nm) and its purity (absorbance ratio 260/230 and 260/280) were measured using a NanoDrop (Thermo Fisher Scientific, Waltham, MA, USA). To ensure the integrity of extracted DNA, it was analyzed by means of gel electrophoresis [19].

### 2.4. Next Generation Sequencing of the Soil Bacterial Community 16s rRNA

The diversity of the total rhizosphere bacterial community was assessed by next generation sequencing (NGS) of amplicons obtained from extracted DNA Sequencing of 16S rRNA amplicons was used for NGS analyses. The first step was to perform a polymerase chain reaction (PCR) with the bacterial primers (linked to the adapter sequences needed for Illumina MiSeq analysis) 341F (5′ TCGTCGGCAGCGTCAGATGTGTATAAGAGACAGCCTACGGGNGGCWGCAG) and 805R (5′ GTCTCGTGGGCTCGGAGATGTGTATAAGAGACAGGACTACHVGGGTATCTAATCC) to amplify the hypervariable regions V3 and V4 of the 16S gene [20]. The thermal conditions were: 95 °C for 3 min, 25 cycles of 95 °C for 30 s; 55 °C for 30 s and 72 °C for 30 s and finally followed by 75 °C for 5 min. All PCR reactions were done using the 1000 Cycler (BioRad, Hercules, CA, USA) thermal cycler.

All the samples consisted of a total volume of 25 μL. This volume consisted of 1 μL DNA (20–60 ng/μL), 12.5 μL KAPA Hifi Hotstart Ready Mix (2.5 mM MgCl_2_, 0.3 mM of each dNTP, KAPA HiFi HotStart DNA Polymerase at 0.5 U per 25 µL reaction) (Roche, Basel, Switzerland); 5 μL (1 µM) of the forward primer, 5 μL (1 µM) of the reverse primer and nuclease free water. To ensure the PCR was successful a 1.5% agarose gel electrophoresis in 1× TAE buffer, containing ethidium bromide (Bio-Rad) was run at 100 V for 30 min.

This was followed by the first PCR clean-up with Agencourt AMPure XP beads (Beckman Coulter Genomics, Chaska, MIN, USA) to purify the amplicons and eliminate free primers and primer dimers. After the first product clean-up, a second PCR with limited cycles was performed that attached dual-index barcodes to the amplicons (Nextera XT Index Kit, Illumina, San Diego, CA, USA) as recommended by the library preparation protocol from Illumina [21]. A second PCR clean-up was performed to clean up the library before quantification. The libraries were quantified with a fluorescence-based method (Invitrogen) using a Qubit 3.0 (Life Technologies, Carlsbad, CA, USA) before normalization and pooling to 4 nM. The pooled library (5 pM) was denatured and 2 × 300 bp paired-end sequencing was conducted with a MiSeq V3 600 cycle reagent cartridge (Illumina) on an Illumina MiSeq according to the manufacturer’s instructions.

### 2.5. NGS Data Bio-Informatics Analysis

Demultiplexing of reads was performed using the on-board MiSeq reporter software (Illumina). The Quantitative Insights into Microbial Ecology 2 (QIIME2) pipeline [22] was used for the processing of NGS data. The quality of reads was evaluated and filtered with demux for elimination of random sequencing errors, deletion of unreliable data from the libraries and removal of reads shorter than 200 bp. Based on the quality control parameters for DADA2, sequences were adjusted and forward and reverse reads assembled. The assembled reads were classified into amplicon sequence variants (ASV) using the feature classifier from QIIME2 software.

The processed sequences were aligned against the SILVANGS rRNA database (SILVA 132release) [23] for taxonomic assignment. The generated ASV count table was summarized in QIIIME2. Graphs of statistically significant bacteria were done using STAMP [24]. Metagenassist was used to do taxonomic to phenotype mapping [25]. MicrobiomeAnalyst was used to do abundance analysis between various stages [26,27]. Using this online tool, alpha diversity was produced using the Chao1 and Shannon diversity indices. With regards to beta-diversity, results were generated using the Bray-Curtis dissimilarity distance distribution. In order to detect the genera with significant differential abundance among the sample fields, linear discriminant analysis (LDA) Effect size (LEfSe) was used [28].

### 2.6. Statistical Analysis of Nematode and Microbial Data

Plant-parasitic nematode population assemblages extracted from the six 20 g composite root samples per field, were pooled and the frequency of occurrence, mean population density (MPD) and prominence value (PV) of each nematode genus calculated [15,29]. Frequency of occurrence was calculated as: (number of localities at which the genus occurred in the root and soil sub-samples of each cultivar/number of localities sampled) × 100. To determine the mean population density (MPD) at each field the total number of individuals of a genus present in root samples of each field was divided by the number of localities in which the genus occurred in root samples. Finally, to determine the prominence value (PV) the mean population density of each genus was multiplied by the √frequency of occurrence and divided by 100. Density classification for *Meloidogyne* and *Pratylenchus* were done as follows: low *x* ≤ 600; medium 601 ≤ *x* ≤ 2999 and high *x* ≥ 3000 (*x* = individuals per 20 g roots).

The alpha diversities of microbial communities, reflected by the bacterial abundance and diversity with regards to the population densities of *Meloidogyne* and *Pratylenchus* individuals in 20 g of soybean roots for each field, were demonstrated using Chao1 boxplots (abundance of bacterial ASV) and Shannon boxplots (community richness). A high Chao1 index indicates a high level of species richness, while a high Shannon index indicates a high level of diversity. Non-metric multidimensional scaling (NMDS) diagrams were used to show the differences between the various rhizosphere microbial communities, beta diversity, of the sample localities. The population densities of *Meloidogyne* and *Pratylenchus* were based on the number of individuals per 20 g of roots. Since the diversity of bacterial communities in each of the fields has its own unique taxonomic abundance profile, the fields with similar taxonomic profiles will group together. Similarities or differences in taxonomic profiles were determined by the Bray-Curtis dissimilarity distance distribution which uses read counts of the bacterial communities. Fields that are plotted close to zero indicate similar taxonomic abundance profiles, whereas sites that don’t plot close to zero have different taxonomic profiles.

Differences in bacterial genus abundance with regards to the population densities of *Meloidogyne* and *Pratylenchus* were evaluated using the LEfSe algorithm [30]. The LEfSe was done using the following parameters: an LDA score of 1 and a cut off *p*-value of 0.05. To determine the link between the abundance of the rhizosphere bacteria that were identified using LEfSe and nematode densities, a functional response model described by Holling [31] was used. The abundances of *Meloidogyne* and *Pratylenchus*, respectively, were compared to the sequence read count (SRC)of each individual bacterial genus ASV to determine whether an increase in the abundance of a certain bacterial genus might cause a decrease in the abundance of the respective said nematode genera. A Windows-based program (CANOCO version 5, Microcomputer Power, Ithaca, NY, USA) was used to generate the response graphs.

## 3. Results

### 3.1. PPN Associated with Soybean Roots

Eleven PPN genera were identified, while those individuals that could not be identified to genus level were listed as belonging to the Order Tylenchida and/or the families Aphelenchoididae and Criconematidae (Table 1). The highest number of nematode genera (9) were present at S9 and S15, while S2 had the lowest number of nematode genera (three) present (Table 1). Of the 12 genera present across the 15 fields, only *Meloidogyne* and *Pratylenchus* were present in each of these localities (Table 1). The highest number of *Meloidogyne* spp. (Table 1) was present at S7 (24,402 individuals/20 g of roots), with S14 having the lowest (183 individuals per 20 g of roots).

With regards to *Pratylenchus* spp., S18 (7851 individuals/20 g of root) and S11 (107 individuals/20 g of root) had the highest and lowest levels, respectively. The PV the nematode genera in all 15 fields ranged from 7 (*Ditylenchus*) to 5291 (*Meloidogyne*) (Table 2). The MPD of *Meloidogyne* were the highest (5291) with *Ditylenchus* and Tylenchida both having the lowest MPD of 28 (Table 2). Some of the other nematode genera present in root samples from the Highveld region were observed in only a few fields. These were *Tylenchorhynchus*, *Ditylenchus*, *Rotylenchus*, *Tylenchus* and nematodes belonging to the Order Tylenchida, and the individuals of the Aphelenchidae and Criconematidae families. Individuals belonging to the genera *Tylenchus* and *Tylenchorhynchus* as well as those identified as belonging to the Criconematidae family are usually ectoparasitic [32] and were potentially feeding actively on the roots when sampling and extractions were done.

### 3.2. Rhizosphere Bacterial Community Associated with Soybean

#### 3.2.1. Alpha Diversity

**Figure 3:**
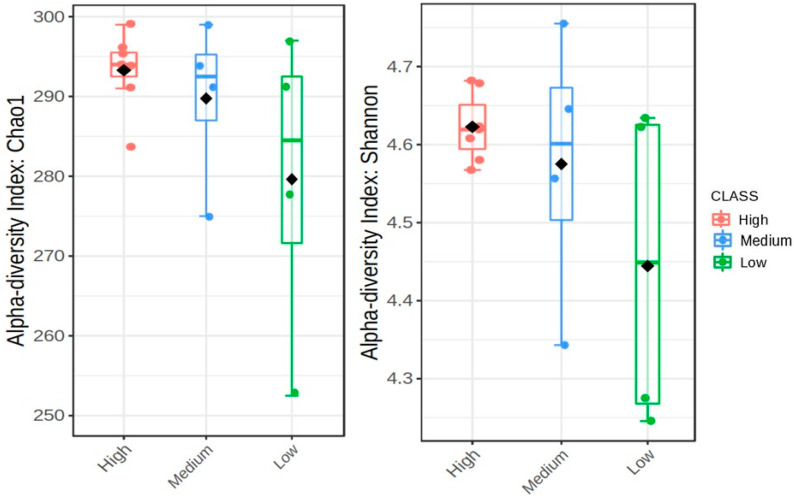
The alpha diversities with regards to *Meloidogyne* infection in soybean roots from the Highveld production area (Mpumalanga province) in South Africa presented as boxplots. The data was plotted with the Chao1 (*p* = 0.211) and Shannon (*p* = 0.170) diversity indices with *p* < 0.05; the median as well as highest and lowest values are indicated on each boxplot.

**Figure 4:**
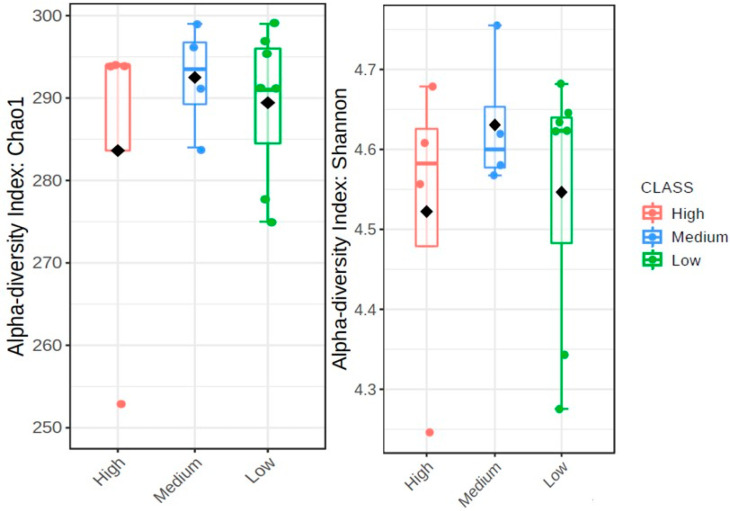
The alpha diversities with regards to *Pratylenchus* infection in soybean roots from the Highveld production area (Mpumalanga province) in South Africa presented as boxplots. The data was plotted with the Chao1 (*p* = 0.614) and Shannon (*p* = 0.592) diversity indices with *p* < 0.05; the median as well as highest and lowest values are indicated on each boxplot.

The boxplots in Figure 3 and Figure 4 represent the alpha diversities which are reflective of the bacterial abundance and diversity of *Meloidogyne* and *Pratylenchus* individuals in 20 g of soybean roots for each field (Table 1). In Figure 3 the Chao1 index reveals that sites with high levels of *Meloidogyne* had higher levels of bacterial ASV abundance (±290–300), whereas sites with low levels of *Meloidogyne* had the lowest bacterial ASV abundance. Yet, when compared to the Chao1 index of Figure 4, it is evident that sites with lower *Pratylenchus* densities had higher levels of bacterial ASV abundance, while sites with higher *Pratylenchus* densities had the lowest bacterial ASV abundance. With regards to the species diversity (Shannon index), Figure 3 showed that sites with the highest *Meloidogyne* densities had less diverse bacterial communities (±4.56–4.69) as compared to those with low and medium densities of *Meloidogyne*. In the case of *Pratylenchus*, (Figure 4) it was evident that sites with medium densities of *Pratylenchus* showed higher bacterial diversity (±4.56–4.75) compared to those with high and low densities of *Pratylenchus.*

#### 3.2.2. Beta Diversity

**Figure 5:**
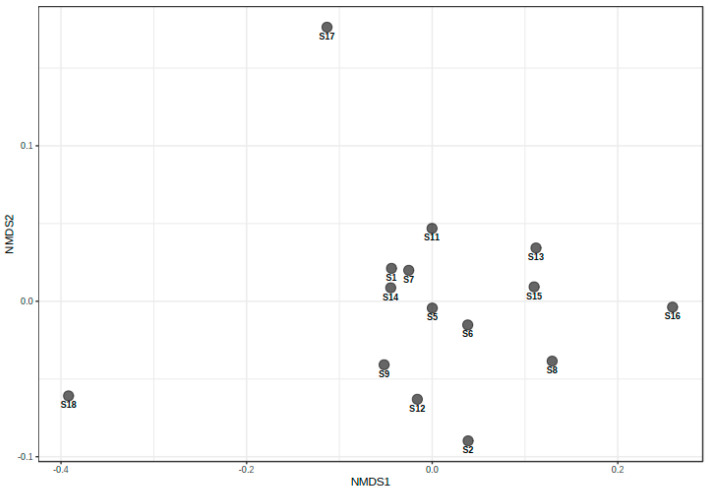
The NMDS diagram shows the beta-diversity of microbe communities among 15 soybean fields sampled from the Highveld region, Mpumalanga province, South Africa. The statistical method used to analyze group similarities was ANOSIM (*p* < 0.85) and applied a Bray-Curtis dissimilarity distance distribution with the sample sites using a correction of R = −0.12262.

The non-metric multidimensional scaling diagram (Figure 5) show the differences between the various rhizosphere microbial communities of the fields sampled. From Figure 5 it is evident that the following fields, S17 and S18, did not group with the others resulting in each of these fields having a different taxonomic microbe profile when compared to those of the other fields. Most of the fields grouped relatively close to zero on both the *x*- and *y*-axis, indicating that they share similar bacterial taxonomic profiles.

#### 3.2.3. Bacterial Populations Associated with Soybean

**Figure 6:**
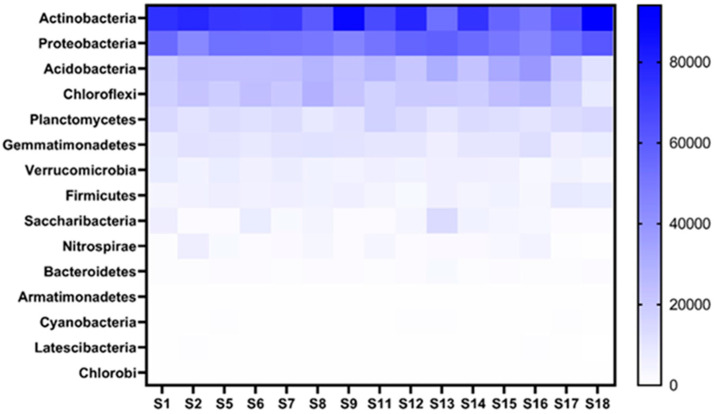
Heatmap indicating the top bacterial phyla and their abundance associated with the soybean rhizosphere of the 15 fields (S1–S18) sampled from the Highveld region, Mpumalanga province, South Africa. The darkness of the blue color indicates the abundance, with darker colors being more abundant.

All the NGS sequences obtained for bacterial populations from soil collected from the 15 fields sampled could be divided into 15 phyla (Figure 6), 47 classes, 55 orders, 91 families and 148 genera. Actinobacteria (33.88%) was the most abundant phyla across the 15 fields followed by Proteobacteria (25.14%). The least abundant phyla were Cyanobacteria (0.11%) Latescibacteria (0.09%) and Chlorobi (0.02%).

**Figure 7:**
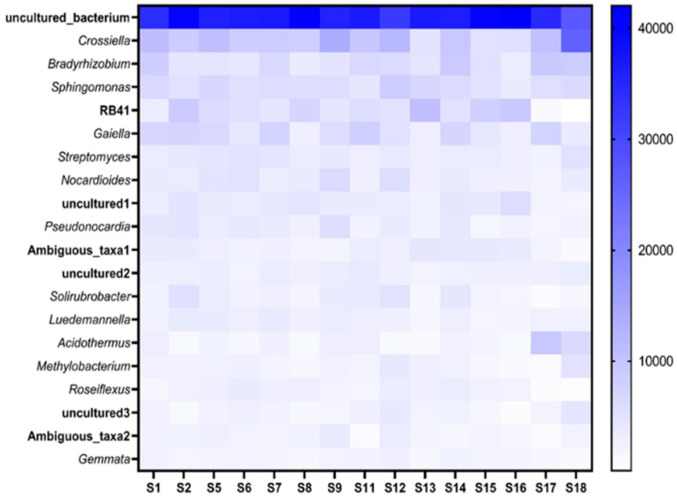
Heatmap indicating the top 20 bacterial genera and their abundance associated with the soybean rhizosphere of the 15 fields (S1–S18). The darkness of the blue color indicates the abundance, with darker colors being more abundant.

Figure 7 indicates the top 20 genera present across all fields. Of these genera uncultured_bacteria and *Crossiella* had the highest abundance across all the fields. Some genera such as *Bradyrhizobium*, *Sphingomonas* and *Acidothermus* amongst others could be identified while there were many genera that could not be identified with the database used and were listed as uncultured. Of the 148 genera identified, all the similar genus names were assigned a number to identify which of these uncultured bacterial genera is being referred to in further analysis.

#### 3.2.4. Linear Discriminant Analysis (LDA) Effect Size (LEfSe)

A total of 9 bacterial genera (Table 3) were found to be significantly more abundant in the soybean rhizospheres of plants sampled for this study. With regards to *Meloidogyne* densities, the genera *Bacillus*2 (*p* = 0.01) and uncultured15 (*p* = 0.03) had significantly higher abundances in fields with medium densities of *Meloidogyne* (Table 1). However, the genera *Gemmata*1 (*p* = 0.02), *Streptomyces*2 (*p* = 0.04), *Roseiflexus*2 (*p* = 0.034), *Pirellula*3 (*p* = 0.034) and Ambiguous_taxa10 (*p* = 0.007) had significantly higher abundances in fields with low densities of *Pratylenchus*. Moreover, the genera uncultured15 (*p* = 0.025) and uncultured30 (*p* = 0.0355) as well as Ambiguous_taxa16 (*p* = 0.026) were significantly more abundant in fields with medium densities of *Pratylenchus*.

### 3.3. Potential Link between Significantly Abundant Rhizosphere Bacteria and PPN Population Density

**Figure 8:**
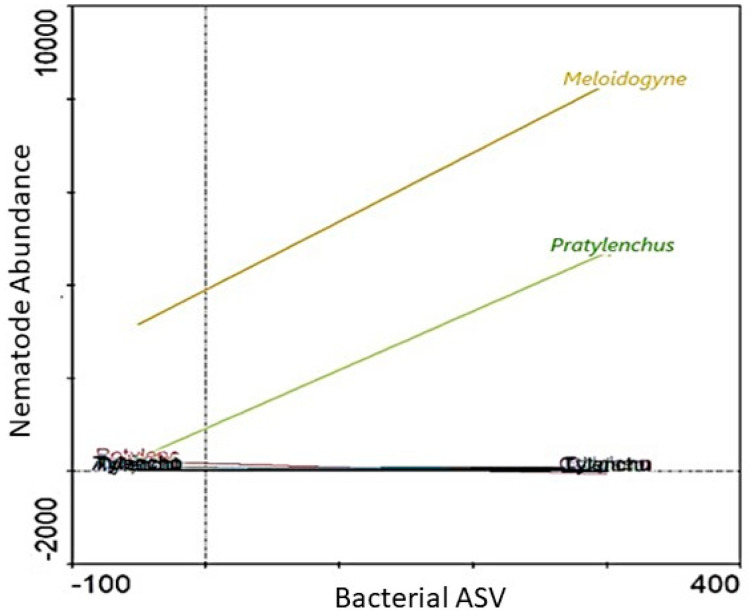
A functional response graph showing the correlation between the abundance (Bacterial ASV) of the genus Ambiguous_taxa16 and the abundance of *Meloidogyne* (orange line) and *Pratylenchus* (green line) that were extracted from soybean roots sampled from the Highveld production area in the Mpumalanga province, South Africa.

**Figure 9:**
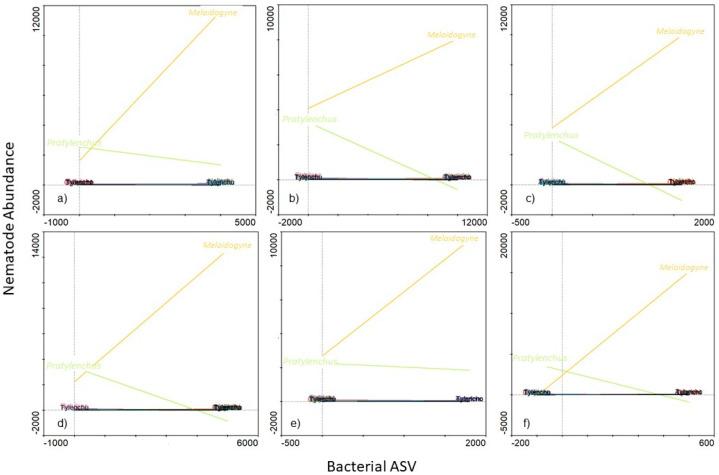
A functional response graph showing the correlation between the abundance (Bacterial ASV) of the genera Bacillus2 (**a**), Gemmata1 (**b**), Pirellula3 (**c**), Streptomyces2 (**d**), uncultured15 (**e**), uncultured30 (**f**) and the abundance of *Meloidogyne* (orange line) and *Pratylenchus* (green line) that were extracted from soybean roots sampled from the Highveld production area in the Mpumalanga province, South Africa.

**Figure 10:**
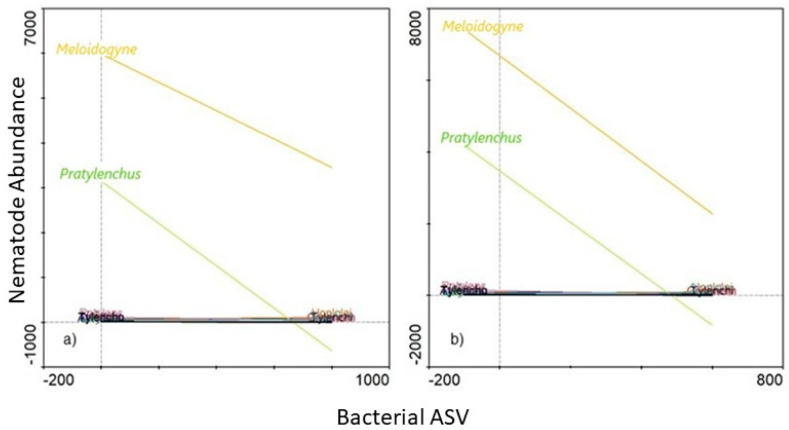
A functional response graph showing the correlation between the abundance (Bacterial ASV) of the genera Ambiguous_taxa10 (**a**), *Roseiflexus*2 (**b**) nematode abundance of the abundance of *Meloidogyne* (orange line) and *Pratylenchus* (green line) that were extracted from soybean roots sampled from the Highveld production area in the Mpumalanga province, South Africa.

Of the 9 bacterial genera that were identified using LEfSe, only Ambiguous_taxa16 (Hyphomicrobiaceae family) was associated with high abundance of both *Meloidogyne* and *Pratylenchus* (Figure 8). In fact, where a high abundance of the Ambiguous_taxa16 (±300 ASV) was evident, both *Meloidogyne* (±8000 individuals) and *Pratylenchus* (±4000 individuals) abundances were also high. Furthermore, from the 9 genera identified using LEfSe, high abundances (SRC) of 6 were associated with low *Pratylenchus* densities (Figure 9). However, of these 6 genera, a high abundance (SRC) of uncultured15 was associated with the lowest *Pratylenchus* abundance. Although the high abundance of these bacterial genera correlated with high *Meloidogyne* densities, high abundance of in *Gemmata*1 (Figure 9b) were inversely correlated with *Meloidogyne* densities. An SRC count of ±10,000 for *Gemmata*1 was associated with ±8000 *Meloidogyne* individuals (Figure 10b), compared to an SRC count of ±5000 for both *Bacillus*2 (Figure 9a) and *Streptomyces*2 (Figure 9d), associated with ±12,000 *Meloidogyne* individuals.

The two remaining genera Ambiguous_taxa10 (Figure 10a) and *Roseiflexus*2 (Figure 10b) were the only two of which high abundances (SRC) were associated with low densities of both *Meloidogyne* and *Pratylenchus*. Of these two genera, high SRC of *Roseiflexus*2 (Figure 10b) were associated with the lowest *Meloidogyne* densities. A *Roseiflexus*2 SRC count of ±600 was, for example, associated with *Meloidogyne* densities of ±2000 individuals. In comparison Ambiguous_taxa10 with an SRC count of ±800 was associated with *Meloidogyne* densities of ±3500 individuals. In both cases of Ambiguous_taxa10 (Figure 10a) and *Roseiflexus*2 (Figure 10b), low *Pratylenchus* densities were found to be associated with increased abundances of these genera.

## 4. Discussion

The current study reports a similar number of PPN genera associated with soy-bean roots (11) than the those reported in 2020 [33] and more than the 7 genera previously reported in 2001 to be associated with soybean in South Africa [15]. This can likely be explained by the improved, adapted methods used for PPN extraction from soybean roots—during this study the protocol of Swart and Marais [17] was used. Also, the expansion of soybean production compared to the beginning of the century when a previous study [15] was done could add to the explanation of this phenomenon. However, when results from this study were compared to the results from a previous study [15], the predominant endoparasites were still found to be *Meloidogyne* and *Pratylenchus* spp. on both occasions. One of the most important observations that was made is the high PV of both the *Meloidogyne* and *Pratylenchus* genera (Table 2). The PV of *Meloidogyne* (Table 2) was higher when compared to that reported by previous studies [15,34]. Crop rotation used in the Mpumalanga Highveld region, especially in the fields used in this study, usually include soybean rotated with grain crops such as maize (*Zeae mays* L.) [35], which is also susceptible RKN. This rotation practice therefore contributes to aggravated strain being placed on the sustainable crop production of grain and legumes in this region.

Moreover, other factors that are not known to the authors could have impacted on the higher PVs of these two genera in the Highveld region compared to those of the 2001 study. With regards to the PV of *Pratylenchus* (lesion nematode), this study reports similar findings to that of Mbatyoti [34]. Although *Pratylenchus* was not considered to be an important pest of soybean [36], recent studies have found that *Pratylenchus* spp. severely impact soybean, causing potential losses of up to 85% in some cases [34,37]. The high PV of *Pratylenchus* in this study might also be caused by the rotation practices used. It has been reported that rotation of soybean with maize in Brazilian production areas, favored the reproduction of *P. brachyurus* [37] and this might have similar effects in South African production areas, such as the Mpumalanga Highveld. Moreover, the common practice of using maize and grain legumes in rotation in the Mpumalanga Highveld will therefore contribute to higher RKN and lesion nematode population densities since these crops have been found to all being susceptible to the two predominant nematode pest genera [10,35]. The impact of climate change is another factor that should be considered in terms of higher abundance of the two predominant endoparasitic nematode genera found in Mpumalanga Highveld study since combined changes in temperature and moisture is for example factors that will and is foreseen to impact on plant-parasitic nematode abundance [38]. Due to high population densities of both RKN and lesion nematodes in soybean roots, more studies are needed that are aimed at the impact of the co-occurrence of these harmful PPNs on soybean yields. The use of poor-host or resistant cultivars of soybean and rotation crops has until recently generally been the only way to reduce PPN numbers in local farmer’s fields. Such a strategy requires that commercially available cultivars are annually screened for their host status to target nematode pest species and that resistance to such pests be introgressed into high yielding genetic material to develop high levels of resistance. This is, however, not receiving priority and therefore this approach cannot be used optimally. Furthermore, Velum 1GR (a.i. fluopyram) has recently been officially registered for use on soybean in South Africa representing the only nematicide available for use by producers [39]. An alternative and/or supplementary approach to the non-optimal use of poor-host or resistant cultivars and limited chemical control options can be the use of endemic biological control agents such as bacteria or fungi. However, management of PPN remains difficult [40]. Although chemicals remain the most common method for RKN management [41], many have elevated levels of toxicity contributing to environmental and human safety concerns. Various chemical nematicides are also increasingly being removed from international markets [42]. This calls for the urgent development of more environmentally friendly PPN control methods.

Previous studies have shown that factors such as the crop that was planted, soil type and the root exudates of the cultivated plants can affect the bacterial community structure of the rhizosphere microbiome by changing the physical and chemical properties of soil [43,44,45]. Although the alpha diversity of rhizosphere bacterial communities with regards to either *Meloidogyne* or *Pratylenchus* were found to be different, the beta diversity (Figure 5) and therefore, the taxonomic profile of the fields was relatively similar. Only the taxonomic profiles of S18 and S17 were different compared to those of the other localities. The difference in the taxonomic profile of S18, when compared to other localities, might be caused by the monocropping of soybean at this locality. Yet, S17 also had a different taxonomic profile, although the reason for this might not be attributed to monocropping like that of S18, but to other complementary and unknown factors warranting further analysis. However, although the differences in the alpha diversity observed in this study were not significantly different, it might be caused by several factors including the root exudates of soybean plants. Root exudates are known to influence rhizosphere bacterial assemblages [43]. As the soybean plants respond to varying PPN densities, the root exudates can act as attractants/stimulants as well as inhibitors/repellents, which may have a profound effect on rhizosphere bacteria potentially causing the observed differences in bacterial diversity and richness [46].

The phyla that were identified in this study were very similar to those reported [47] when examining the soybean rhizosphere in Kyoto, Japan and that of soybean fields across China [48]. However, in these studies Proteobacteria was identified as the most abundant phylum. *Bradyrhizobium*, *Sphingomonas*, *Bryobacter* and *Streptomyces* were identified from the top 20 listed genera in the soybean rhizosphere in two similar studies [33,49]. There are microorganisms present in the soil that are not pathogenic towards plants and of the bacterial genera identified in Figure 8, *Bradyrhizobium* and *Sphingomonas* are examples of these. *Bradyrhizobium*, a nitrogen-fixing symbiont of legumes, would usually be abundant in higher numbers when analyzing the rhizosphere of legumes [50] and explains the high abundance of this genera reported in this study. The plant growth-promoting endophytic bacteria (PGPEB) *Sphingomonas* can occur in diverse environments. Together with its plant growth promoting capabilities, this genus can also decompose various pesticides such as those that contain the active ingredient cypermethrin [51,52]. Other reports suggest that bacteria belonging to the genera *Methylobacterium* have been found in soils that are suppressive against the genus *Meloidogyne* in vegetable production sites in Grossbeeren, south of Berlin, Germany [53] and sites with a history of RKN infestation in Spain [54]. This corresponds with the abundance of *Methylobacterium* identified in the localities investigated in this study. The *Bacillus* genus has been associated with the soybean rhizosphere and promotes its plant growth [55] as well as being present in soils with low densities of *P. neglectus* and *M. chitwoodi* in potato farms of the San Luis Valley, Colorado, USA [56]. Other genera such as *Gemmata*, *Streptomyces* and *Roseiflexus* have also been reported from the rhizosphere of soybean fields in Kyoto, Japan and the Heilongjiang Province of China [48,57]. Furthermore, although a previous study found that bacteria belonging to genera such as *Lysobacter*, *Steroidobacter*, *Flavobacterium*, *Chryseobacterium* and *Flexibacter* were present in soils with low densities of *Meloidogyne*, none of these genera were identified as significant in this study [54].

Several studies have reported the presence of the *Gemmata* genus [58] in environments ranging from bogs in Russia [59], a compost heap in Northern Germany [60] as well as a water spring in South Africa [61]. Although these studies did not aim to study the nematicidal potential of this genus, a study done [62] found that *Gemmata obscuriglobus* is capable of polyketide and non-ribosomal peptide synthesis. These compounds can activate plant defenses and contribute to a potential decrease in nematode infections [63,64]. In a study done in China [47], they compared the rhizosphere of soybean and another legume plant, alfalfa (*Medicago sativa*) and found that the genus of the Planctomycetes phylum, *Pirellula* to be more abundant in the rhizosphere of alfalfa than that of soybean. A strain of this genus, also known as *Rhodopirellula*, has been identified in the soybean root endosphere. This genus was found to be present in soybean monoculture systems in north-eastern China with suppressive effects against the soybean cyst nematode, *Heterodera glycines* [65]. It is possible that a higher abundance of several bacterial genera, such as those mentioned above, might cause reduced levels of parasitic nematodes like *Meloidogyne* and *Pratylenchus*. The identification of such bacterial genera and their abundance will therefore provide valuable information regarding bacteria that might be used as potential biocontrol agents in nematode management.

It has been reported that the *Streptomyces* genus has high abundances in the soybean rhizosphere [66]. This genus has been reported to be suppressive against *Fusarium* wilt disease [67] as well as the soybean cyst nematode, *Heterodera glycines* [68] and the RKN, *M. incognita* [69,70]. A novel strain belonging to the *Streptomyces* genus was also isolated from nematode-suppressive soil in Costa Rica [71]. Furthermore, *Streptomyces* spp. were found to have suppressive effects against the lesion nematode, *P. penetrans* that parasitizes alfalfa in Minnesota and Wisconsin field soils [72]. In the case of *Bacillus*2 (Figure 9a), this genus belongs to the family Bacillaceae. Various species of the genus *Bacillus* has been known to have nematicidal activity against harmful nematode pests such as *Meloidogyne* spp., *Pratylenchus* spp. and *Heterodera* spp. Amongst these are *B. pumilus*, *B. megaterium*, *B. thuringiensis* and *B. soli* [69,73,74,75,76].

Ambiguous_taxa10 belongs to the Hyphomicrobiaceae family, which has been identified in soybean monoculture systems in Minnesota, USA [77], with the genus *Rhodoplanes* (Hyphomicrobiaceae family) identified in potato farms of the San Luis Valley (Colorado, USA). However, *Rhodoplanes* was found to be positively correlated with *M. chitwoodi* in a previous study. Yet, our results suggest that the abundance of the Ambiguous_taxa10 genus, belonging to the Hyphomicrobiaceae family, shows a negative correlation with relation to both *Meloidogyne* and *Pratylenchus* densities, contrasting results previously reported [56]. The relation of *Roseiflexus*2 (Figure 10b) abundance towards *Meloidogyne* and *Pratylenchus* densities proves quite interesting, as this genus has been found to be related to uncultivated filamentous phototrophic bacteria, predominately present in microbial mats of hot springs [78]. To our knowledge, there has not been any reports of the potential nematicidal activity of this genus and future studies will thus generate novel information in this regard.

## 5. Conclusions

Plant-parasitic nematodes cause extensive losses to various economically important crops in South Africa, including soybean. Notably, most research has been done on species of PPN genera such as *Heterodera*, *Meloidogyne* and *Pratylenchus* and their potential impact on soybean production. There are various control strategies such as nematicides, both chemical and biological, that can be used to manage the impact of the PPN. However, more research is being done on the use of microorganisms as potential biocontrol agents of nematodes to fill the gap left by the removal of various chemical nematicides from the international markets. Research relating to biocontrol remains challenging as the nematicidal effects observed for microbes in in vitro studies often fail to reproduce upon the reintroduction of these strains into field studies [79]. While the identification of bacterial strains with nematicidal activity in vitro remains helpful, DNA based classification of the microbiomes associated with the natural rhizosphere of soybean plants with low PPN densities can provide a more comprehensive understanding of bacteria with nematicidal activity in such an environment. Since less than 1% of bacterial spp. can be cultivated in a laboratory [80,81], 16S rRNA gene amplification and more recently NGS have emerged as powerful tools that can be used to study microbial populations [81]. Even so, identification of genera still proves difficult, resulting in numerous genera not being identified. A possible explanation for the observations made in this study, with regards to bacterial ASV as well as *Meloidogyne* and *Pratylenchus* densities might be a result of competition between organisms (including both bacteria and nematodes). This is caused by the environmental conditions or mixtures of different bacteria having various nutritional and environmental requirements that influences certain metabolic capabilities of these bacteria [82,83], potentially causing changes in their nematicidal activity. In a similar study [57], the authors concluded that a consortium of bacteria with nematicidal properties can exist on a spatial scale within a field of soybean that is infected by RKN. There could then be a possibility of identifying several biological control agents that are potentially available in situ without introducing any “foreign” bacterial strain(s). Improving our understanding of the natural rhizosphere bacterial and fungal communities and their relationship with both the plant and nematodes will help unravel the natural microbiome structure needed for biocontrol of PPN.

## Figures and Tables

**Table 1:** The community structure and abundance of plant parasitic nematodes in 20 g soybean root samples collected during the 2018/19 growing season from 15 fields of commercial producers in the Highveld region of the Mpumalanga province of South Africa.

Genus and/or Family	Field No.														
	S1	S2	S5	S6	S7	S8	S9	S11	S12	S13	S14	S15	S16	S17	S18
*Meloidogyne*	344	2548	3625	1141	24,402	4913	4980	7400	999	5097	183	21,757	1059	518	394
*Pratylenchus*	518	9350	550	784	3584	655	1826	107	243	4331	270	1004	229	335	7851
*Helicotylenchus*	44	132	28	28	170	28	248	87	28	28	34	110	83	28	0
*Scutelonema*	87	0	66	66	101	34	83	38	110	41	77	41	41	50	28
*Hoplolaimus*	96	0	37	105	89	28	118	72	96	34	69	65	143	57	0
*Rotylenchulus*	28	0	28	34	60	46	1000	62	37	0	55	41	0	0	0
*Tylenchorhynchus*	0	0	0	0	0	0	37	14	0	0	0	193	0	7	69
*Ditylenchus*	0	0	0	0	28	0	0	0	0	0	0	0	0	0	0
*Rotylenchus*	0	0	0	0	0	0	39	0	0	94	0	0	0	0	37
*Tylenchus*	0	0	0	0	0	28	55	0	28	0	0	0	0	0	46
Tylenchida	0	0	0	0	0	0	0	0	28	0	0	28	0	0	0
Aphelenchidae	28	0	28	0	0	0	0	0	0	28	0	97	0	0	0
Criconematidae	0	0	0	28	0	0	0	14	0	0	0	0	28	0	55
Level of *Meloidogyne* infection	Low	Medium	High	Medium	High	High	High	High	Medium	High	Low	High	Medium	Low	Low
Level of *Pratylenchus* infection	Low	High	Low	Medium	High	Medium	Medium	Low	Low	High	Low	Medium	Low	Low	High

**Table 2:** Prominence values, frequencies of occurrence and mean population densities of plant parasitic nematode genera occurring in 20 g soybean root samples collected during the 2018/19 growing season from 15 fields of commercial producers in the Highveld region of the Mpumalanga province of South Africa.

Genus and/or Family	^b^ Mean Population Density (MPD)	^a^ Frequency of Occurrence (FO)	^c^ Prominence Value (PV)
*Meloidogyne*	5291	100	5291
*Pratylenchus*	2109	100	2109
*Helicotylenchus*	77	93	74
*Scutelonema*	62	93	60
*Hoplolaimus*	78	87	72
*Rotylenchulus*	139	67	113
*Tylenchorhynchus*	64	33	37
*Ditylenchus*	28	7	7
*Rotylenchus*	57	20	25
*Tylenchus*	39	27	20
Tylenchida	28	13	10
Aphelenchidae	45	27	23
Criconematidae	31	27	16

^a^ FO = (Number of samples containing genus/number of samples collected) × 100. ^b^ MPD = total number of individuals of a genus present in root samples of each site/number of localities in which the genus occurred in root samples of each site. ^c^ PV = MPD × √absolute frequency)/100.

**Table 3:** Classification of bacterial genera that were significantly more abundant in the rhizosphere of the 15 soybean fields (sampled from the Highveld region, Mpumalanga province, South Africa) used in this study according to LefSe.

Phylum	Class	Order	Family	Genus
Proteobacteria	Alphaproteobacteria	Rhizobiales	Hyphomicrobiaceae	Ambiguous_taxa10
Proteobacteria	Deltaproteobacteria	Myxococcales	Sandaracinaceae	uncultured15
Firmicutes	Bacilli	Bacillales	Bacillaceae	Ambiguous_taxa16
Planctomycetes	Planctomycetacia	Planctomycetales	Planctomycetaceae	*Gemmata*1
Planctomycetes	Planctomycetacia	Planctomycetales	Planctomycetaceae	*Pirellula*3
Chloroflexi	Chloroflexia	Chloroflexales	Roseiflexaceae	*Roseiflexus*2
Firmicutes	Bacilli	Bacillales	Planococcaceae	uncultured30
Actinobacteria	Actinobacteria	Streptomycetales	Streptomycetaceae	*Streptomyces*2
Firmicutes	Bacilli	Bacillales	Bacillaceae	*Bacillus*2

## Data Availability

Data, referring to those for nematodes and microbes, are available from the principal author.

## Supplementary Materials

The following are available online at https://www.mdpi.com/article/10.3390/microorganisms9091813/s1, Table S1: Details of 15 fields of commercial producers in the Highveld region of South Africa where soybean rhizosphere (root and soil) samples were collected during the 2018/2019 growing season for nematode and microbe analyses.
