# Supplementary material for: Screening of Rhizosphere Bacteria and Nematode Populations Associated with Soybean Roots in the Mpumalanga Highveld of South Africa
Source: Microorganisms. 2021 Aug 26;9(9):1813. doi: 10.3390/microorganisms9091813 (PMC8469482; doi:10.3390/microorganisms9091813)
Supplement: Supplementary file 1 [file microorganisms-09-01813-s001.zip › microorganisms-1327333-supplementary.pdf]

Table S1: Details of 15 fields of commercial producers in the Highveld region of South Africa where soybean rhizosphere (root and soil) samples were collected during the 2018/2019 growing season for nematode and microbe analyses.

Field	GPS Coordinates	Altitude (m)	Crop rotation history prior to 2018/2019
**S1**	25°50'04.4"S, 29°54'02.2"E	1747	Soybean-Maize-Soybean
**S2**	25°49'28.2"S, 29°32'42.6"E	1604	Maize-Maize-Maize
**S5**	26°19'08.7"S, 29°30'27.2"E	1625	Maize-Soybean-Soybean
**S6**	26°14'50.4"S, 29°38'09.7"E	1661	Maize-Soybean-Maize
**S7**	26°14'52.6"S, 29°38'18.8"E	1652	Maize-Soybean-Maize
**S8**	26°16'59.3"S, 29°36'48.4"E	1659	Soybean-Soybean-Maize
**S9**	26°17'10.2"S, 29°36'44.0"E	1661	Soybean-Soybean-Maize
**S11**	25°49'00.3"S, 29°32'56.9"E	1591	Maize-Maize-Maize
**S12**	25°46'43.6"S, 29°38'36.4"E	1631	Maize-Maize-Maize
**S13**	26°17'17.2"S, 29°36'44.8"E	1660	Maize-Soybean-Maize
**S14**	26°12'06.5"S, 30°08'43.6"E	1731	Maize-Soybean-Maize
**S15**	26°12'09.6"S, 30°07'31.2"E	1714	Maize-Maize-Soybean
**S16**	26°29'30.6"S 30°04'38.5"E	1687	Soybean-Maize-Maize
**S17**	26°29'35.7"S 30°05'00.5"E	1677	Soybean-Maize-Maize
**S18**	26°01'43.0"S, 28°48'57.9"E	1518	Soybean-Soybean-Soybean
